# A case report of MYH7 mutation-induced restrictive cardiomyopathy

**DOI:** 10.1093/ehjcr/ytaf166

**Published:** 2025-04-08

**Authors:** Shaozhen Zhang, Wang Zhao

**Affiliations:** Department of Cardiovascular Medicine, The Second Xiangya Hospital, Central South University, No. 139, Middle Renmin Road, Changsha, Hunan 410011, China; Department of Cardiovascular Medicine, The Second Xiangya Hospital, Central South University, No. 139, Middle Renmin Road, Changsha, Hunan 410011, China

**Keywords:** MYH7 gene, Gene mutation, Restrictive cardiomyopathy, Hypertrophic cardiomyopathy, Case report

## Abstract

**Background:**

Restrictive cardiomyopathy (RCM) is characterized by impaired diastolic function and ventricular filling, often due to genetic and environmental factors. The *MYH7* gene, encoding myosin heavy chain in muscle fibres critical for muscle contraction, has been implicated in RCM.

**Case summary:**

We describe the case of a female patient who was presented with recurrent chest tightness and shortness of breath. Based on imagining findings and genetic testing, she was diagnosed with *MYH7*-induced RCM. Her daughter inherited the same variant but presented with a hypertrophic phenotype.

**Conclusion:**

*MYH7*-induced cardiomyopathy is a complex condition, associated with variable clinical presentation and phenotype. While imagining and endomyocardial biopsy play important roles in diagnosing RCM, their application might be limited for economic and safety reasons. Further research is needed to elucidate the pathogenesis and develop safer and cheaper approaches to diagnose *MYH7*-induced restrictive cardiomyopathy.

**Learning points**

- MYH7 mutations follow an autosomal dominant inheritance pattern, so genetic testing is crucial for the early diagnosis of restrictive cardiomyopathy (RCM).
- Endomyocardial biopsy is important in diagnosing RCM, as it reveals indicative pathological changes like myofibre disarray, patchy interstitial fibrosis, and myocyte degeneration.

## Introduction

Restrictive cardiomyopathy (RCM) presents as an impaired diastolic function and ventricular filling disorder, attributable to genetic susceptibility and environmental factors. While the ejection fraction may initially remain normal, progression can lead to a diminished ejection fraction and heart failure.^1^ The myosin heavy chain 7 gene (*MYH7*) is one of the genes encoding myosin heavy chain that participates in the contraction process of muscle as molecular motors.^2^ While its mutation is well-established as a common genetic cause of hypertrophic cardiomyopathy (HCM), the association of *MYH7* mutations with RCM is exceptionally rare, with fewer than 10 cases reported worldwide.^1^ Influenced by abnormal protein, the endocardium undergoes extensive fibrosis, which is the most prominent in the atria, and causes varying degrees of atrial dilation without obvious myocardial hypertrophy.^3^ However, the mechanisms involved remain poorly understood, and limited case studies have been reported. Herein, we report a rare case of RCM caused by a variation in *MYH7* expression.

## Summary figure

**Figure:**
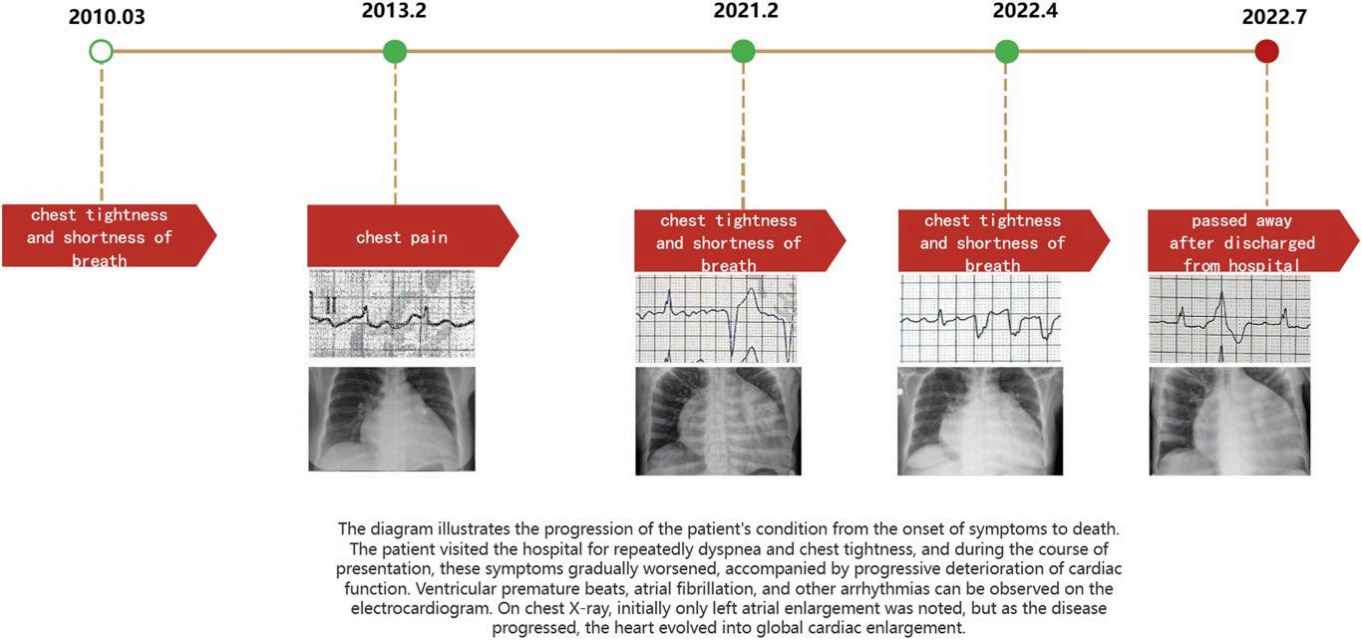


## Case presentation

**Figure 1:**
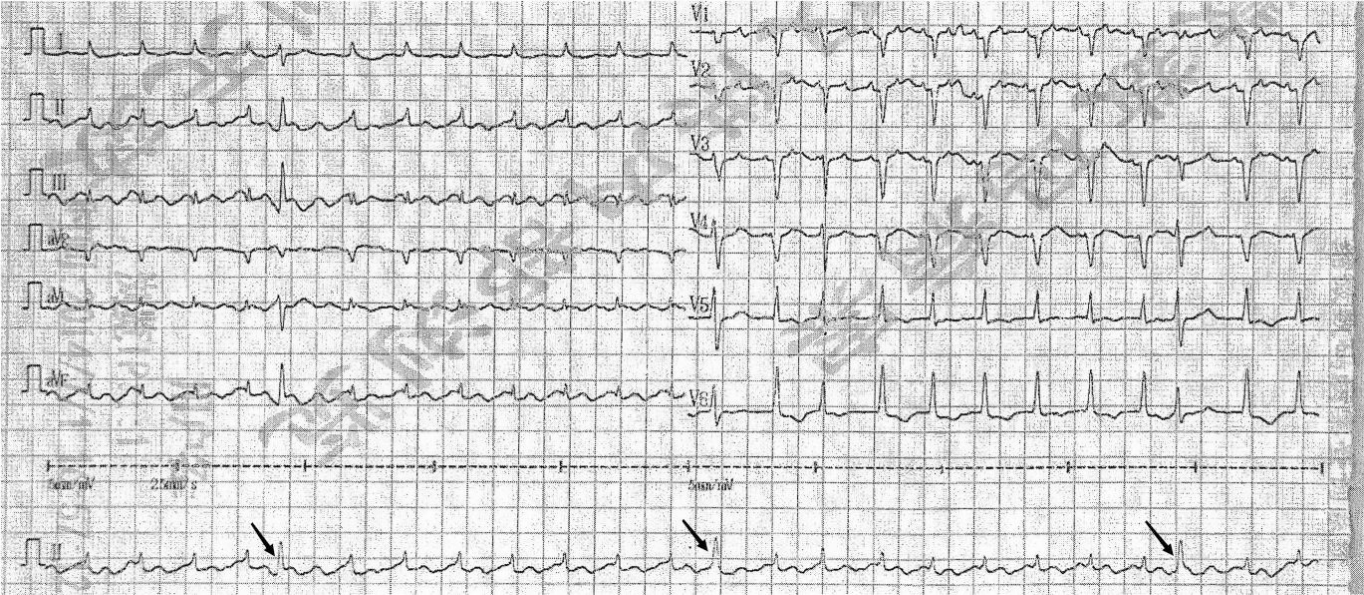
ECG in February 2013. Frequent premature ventricular contractions can be seen in this picture. The P waves disappeared in all leads, replaced by atrial flutter waves, suggesting atrial flutter.

**Figure 2:**
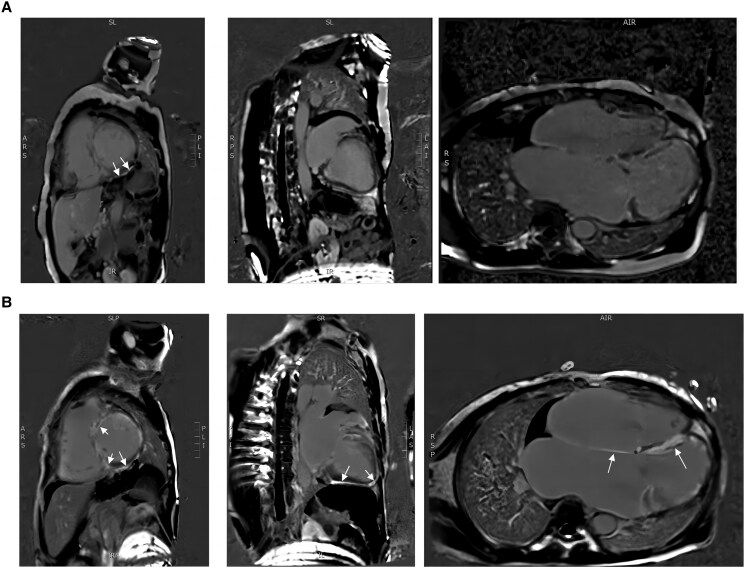
(*A*) Cardiac MRI of the patient in 2021. (*B*) Cardiac MRI of the patient in 2022. In the patient's cardiac MRI in 2021 (*A*), late gadolinium enhancement imaging of the myocardium revealed delayed enhancement of the interventricular septum and the epicardium of the left ventricular inferior wall. However, in 2022, cardiac MRI showed widespread delayed enhancement of all walls of the left ventricle, indicating a progression of the disease.

**Figure 3:**
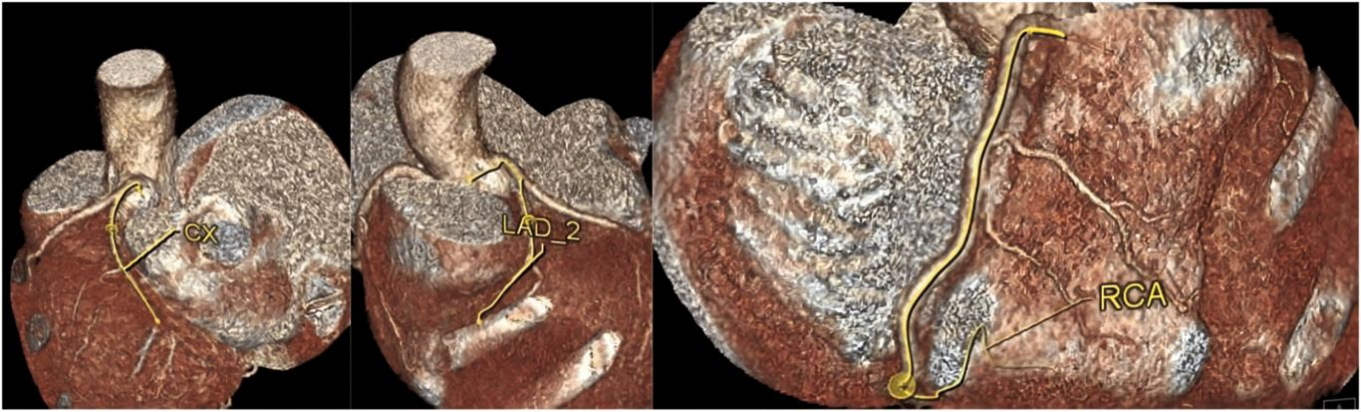
Coronary computed tomography angiography of the patient. As it was shown in the coronary computed tomography angiography, there was no stenosis observed in the left main coronary artery, left anterior descending artery, circumflex artery, or right coronary artery. CCTA, coronary computed tomography angiography; CX, circumflex artery; LAD, left anterior descending artery; RCX, right coronary artery.

**Figure 4:**
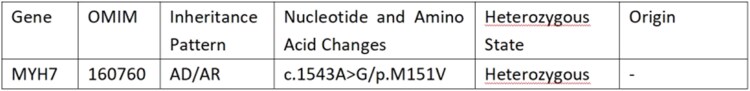
Genetic test result of the proband. The patient had c.1543A > G mutation on gene MYH7. However, it passed as an autosomal dominant or autosomal recessive manner.

A 58-year-old female was admitted to our institution six times over the past 12 years because of recurrent chest tightness and shortness of breath. The echocardiogram (ECHO) done on her first presentation in March 2010 showed a dilated left atrium (44 millimetres) and mild-to-moderate mitral regurgitation (*Table 1*). Therefore, the patient was diagnosed with valvular heart disease and was hospitalized multiple times. Physical examination revealed the presence of rales in the lungs, decreased heart impulse, arrhythmia, and varying intensity of the first heart sound. So, she received symptomatic treatments including warfarin (anticoagulation), metoprolol (heart rate control), irbesartan (anti-ventricular remodeling management), and diuresis. Although she received timely medication, she continued to experience the above symptoms, along with chest pain and bloating. Over time, she gradually developed atrial flutter and frequent premature ventricular contractions (*Figure 1*). Some ST segment changes can be seen in electrocardiography (see Supplementary material online, *S1*). Over the years, serial echocardiograms performed at follow ups and during hospitalizations revealed progressive enlargement of all cardiac chambers, more prominent in the atria, and progressive diastolic and systolic dysfunction (*Table 1*). Cardiac magnetic resonance imaging (CMR) revealed late gadolinium enhancement (LGE) of the interventricular septum and epicardium of the left ventricular inferior wall, consistent with extensive fibrosis of the left ventricle and interventricular septum (*Figure 2A*). Coronary computed tomography angiography revealed no significant coronary stenosis (*Figure 3*). The patient told us that several of the patient's first-degree relatives had a history of ‘heart disease’ and ‘cardiac enlargement’ and died of ‘heart disease’ in middle age (see Supplementary material online, *S2*). Unfortunately, owing to constraints in the available medical technology and institutional resources, we were unable to obtain the patient's family medical records. Due to the special family history, we performed genetic testing and identified a c.1543A > G mutation in the *MYH7* gene (*Figure4A*). This genetic mutation subsequently results in a p.Met515Val change in the myosin heavy chain. She underwent a second CMR examination in 2022. The result revealed a larger fibrotic area (*Figure 2B*). Based on the results of imaging findings and her symptoms, she was diagnosed with MYH7 variation-induced RCM with left ventricular enlargement. In July 2022, the patient was admitted to our hospital for the sixth time due to recurrent chest tightness and shortness of breath over 9 years, worsened by abdominal distension for 1 month. During her stay, she experienced shock and severe heart failure, necessitating transfer to the Cardiac Care Unit for close monitoring and treatment. To further determine pathological phenotype, we suggested to perform endomyocardial biopsy (EMB), but the patient’s family declined due to the associated risks (Throughout the care process, due to the patient's confusion and communication difficulties, as well as rapid deterioration of the condition, the medical team, in accordance with China's ‘Regulations on the Administration of Medical Institutions’ and the relevant provisions of the ‘Civil Code,’ fully communicated with the patient's immediate family members, who exercised medical decision-making authority on behalf of the patient.). The patient was discharged from hospital on 27 July 2022 and soon died because of severe heart failure.

**Table 1:** Changes of cardiac function and structure from 2010 to 2022

	LVD (mm)	LAS (mm)	RVD (mm)	RAS (mm)	LVEF	End diastolic volume (mL)	Velocity of E’ wave (cm/s)	Velocity of E wave (cm/s)	Deceleration time of E wave (ms)
March 2010	46	44	27	27	65%				
September 2012	51	44	25	24	60%				
February 2013	52	51	29	32	60%				
August 2013	56	49	29	34	63%				
October 2020	62	61	48	47	53%				
February 2021	61	51	45	47	42%	201.65	10.0		
April 2022	60	61	35	49	45%	156.2	13.9	133	120
July 2022	56	61	37	51	48%				

From 2010 to 2022, the left and right atrium gradually enlarged and the systolic function rapidly decreased since 2020. As it was shown in the table, the end diastolic volume was reduced in just 1 year, while the velocity of E’ wave increased with a high velocity of E wave. All these evidence above indicated an impaired diastolic function. LAS, left atrium size; LVD, left ventricle diameter; LVEF, left ventricular ejection fraction; RAS, right atrium size; RVD, right ventricle diameter.

Considering that this variation is inherited in an autosomal dominant manner, it was necessary to perform genetic testing for all the patient's first-degree relatives who were alive. Only her daughter was affected (see Supplementary material online, *S3*). The daughter reported that she had shortness of breath, chest tightness, fatigue, and decreased exercise tolerance since 2021. Echocardiogram revealed an enlarged left atrial, incoherent heart movement, and impaired diastolic function. Interestingly, CMR found localized thickening of the apical segment (see Supplementary material online, *S4*). Based on the medical history and examination results of the patient's daughter, she was diagnosed with non-obstructive hypertrophic cardiomyopathy.

## Discussion

*MYH7* encodes the β-myosin heavy chain, which is mainly responsible for binding with actin and ATP to generate force. Thus, the mutation has long been considered closely related to primary cardiomyopathy.^4–7^ Mutations affecting myosin-binding sites may lead to abnormal muscle relaxation and filament structure,^8^ decreasing cardiac output and resulting in symptoms such as chest tightness and shortness of breath. Normally, RCM presents as biatrial enlargement with endocardial hyperplasia and scarring.^1,3^ In rare cases, coronary arteries are surrounded by fibrosis, which causes vessel stenosis and ischemia.^3^ In the early phase of RCM, patients can present normal or nearly normal contractile function and sometimes with cardiac hypertrophy, making it difficult to distinguish patients with RCM from those with HCM.^9^ Thus, multimodality imagining like ECHO, CMR, computed tomography, and nuclear techniques are required to differentiate RCM from HCM.^10^ In ECHO, RCM is primarily characterized by asymmetric septal hypertrophy (≥15 millimetres), accompanied by systolic anterior motion of the mitral valve (SAM sign).^11^Cardiac magnetic resonance imaging serves as a complementary method to echocardiography, providing more comprehensive supplementary information (such as detecting myocardial hypertrophy in areas not visible on ultrasound scans, quantifying left ventricular mass and function, and identifying subtle morphological features of the myocardium, etc.).^11^ Hypertrophic cardiomyopathy typically presents with a restrictive filling pattern (such as an increased E wave and a decreased A wave, elevated E/e’ ratio) and biatrial enlargement, but the ventricles are usually not enlarged or may even be reduced in size.^12^ In some specific subtypes, myocardial hypertrophy, mitral valve thickening, and pericardial effusion may occur.^12^ Myocardial oedema and LGE are usually observed on CMR.^12^ As functional assessments typically show normal or mildly reduced left ventricular ejection fraction, longitudinal systolic function may be impaired in the early stages of the disease.^12^ In this case, the patient presented generalized cardiomegaly with mild decreased ejection fraction (*Table 1*). However, ECHO and CMR revealed impaired diastolic function (*Table 1*) and sever cardiac fibrosis, which align with the imaging features of RCM. Invasive diagnostic methods such as EMB should be considered to exclude specific subtypes of RCM and make a diagnosis.^10^ According to the latest guidelines issued in China, EMB is thought to be irreplaceable in confirming diagnosis, making treatment decisions and assessing prognosis, emphasizing its value in the application of cardiomyopathies.^13^ The most common findings are myofibres disarray, patchy interstitial fibrosis, myocyte degeneration, and sometimes, cardiac hypertrophy.^3,14,15^ In specific subtypes, the histology may show characteristic pathological changes, such as the infiltration of eosinophils and endocardial elastin fibre hyperplasia.^16^ Electron microscopy reveals the structure of myofibrils is disrupted, affecting normal muscle function.^6^ Inflammatory cell infiltration, cell necrosis, and abnormal morphology of organelles have also been observed.^6^ In the reported case, although advanced imaging techniques provided substantial evidence supporting the diagnosis of RCM, EMB offered further histological evidence to confirm the genetic and imagining findings. However, EMB is not risk-free. Because EMB is invasive, it may cause discomfort and anxiety in patients, especially those with advanced heart disease or other comorbidities. In the present case, the patient was on warfarin therapy, which increases the risk of bleeding complications associated with EMB. In addition, the family's refusal to consent for the procedure further limited the feasibility of performing EMB.

The pathogenesis of *MYH7*-induced RCM is complex and has not yet been fully elucidated. One of the possible explanations is that *MYH7* mutations cause imbalance in the expression of intercellular variant/normal proteins, generating inconsistent contractile forces that lead to disordered myocardial arrangement and interstitial fibrosis through TGF-β/Smad signaling pathway.^17,18^ This mutation also causes increased collagen synthesis and deposition, resulting in a higher risk of myocardial fibrosis.^18^

Notably, although the patient and her daughter carried the same mutation, they presented different phenotypes. One possible reason is that the abnormal protein varies in quantity among different patients. Functional changes caused by mutation may alter the biological activity of proteins, causing their deposition in cardiac cells. It also promotes the binding of newly synthesized proteins to other substances, thereby contributing to extracellular matrix synthesis and secretion. Konno *et al*.^19^ found that myosin heavy chain mutation activates the Mef2 family and stimulates cells to re-express foetal genes. These cells then undergo necrosis, and the heart is repaired by fibrous tissue, which ultimately causes ventricular remodelling and wall stiffness. However, Mef2 activation itself does not cause myocardial hypertrophy and is heterogeneous, resulting in different clinical manifestations in each patient.^19^ In addition, changes in protein structure caused by each *MYH7* mutation site are different.^20^

## Patient consent statement

The authors confirm that they have obtained all necessary patient consent forms. Because the patient passed away soon after she was discharged from the hospital, the authors obtain the consent of publishing this article from the patient’s daughter. In the forms, the patient’s daughter has provided consent for their images, case details, and other relevant clinical information to be included in this document. The patient’s daughter understands that their information will be published anonymously, and every effort has been made to protect their identity in accordance with the guidelines set by the Committee on Publication Ethics (COPE).

## Lead author biography


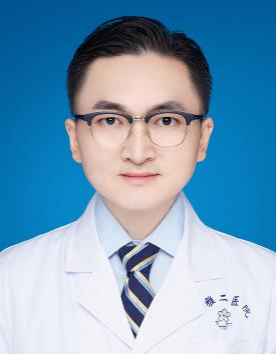


Lead author Zhao Wang has long been engaged in research on blood lipids and atherosclerosis and has extensive experience in the diagnosis and treatment of cardiovascular diseases such as coronary heart disease, dyslipidemia, hypertension, and heart failure.

## Supplementary Material

ytaf166_Supplementary_Data

## Data Availability

The data underlying this article are available in its online Supplementary material.

## Notes

**Consent:** The patient passed away soon after discharging from the hospital. So, we reached out her daughter and got her approval of publishing this case.

**Funding:** This work has been funded by National Natural Science Foundation of China (81500234) and Natural Science Foundation of Hunan Province (2021JJ30980).
