# Supplementary figures and images for: A case report of MYH7 mutation-induced restrictive cardiomyopathy
Source: Eur Heart J Case Rep. 2025 Apr 8;9(4):ytaf166. doi: 10.1093/ehjcr/ytaf166 (PMC12023744; doi:10.1093/ehjcr/ytaf166)

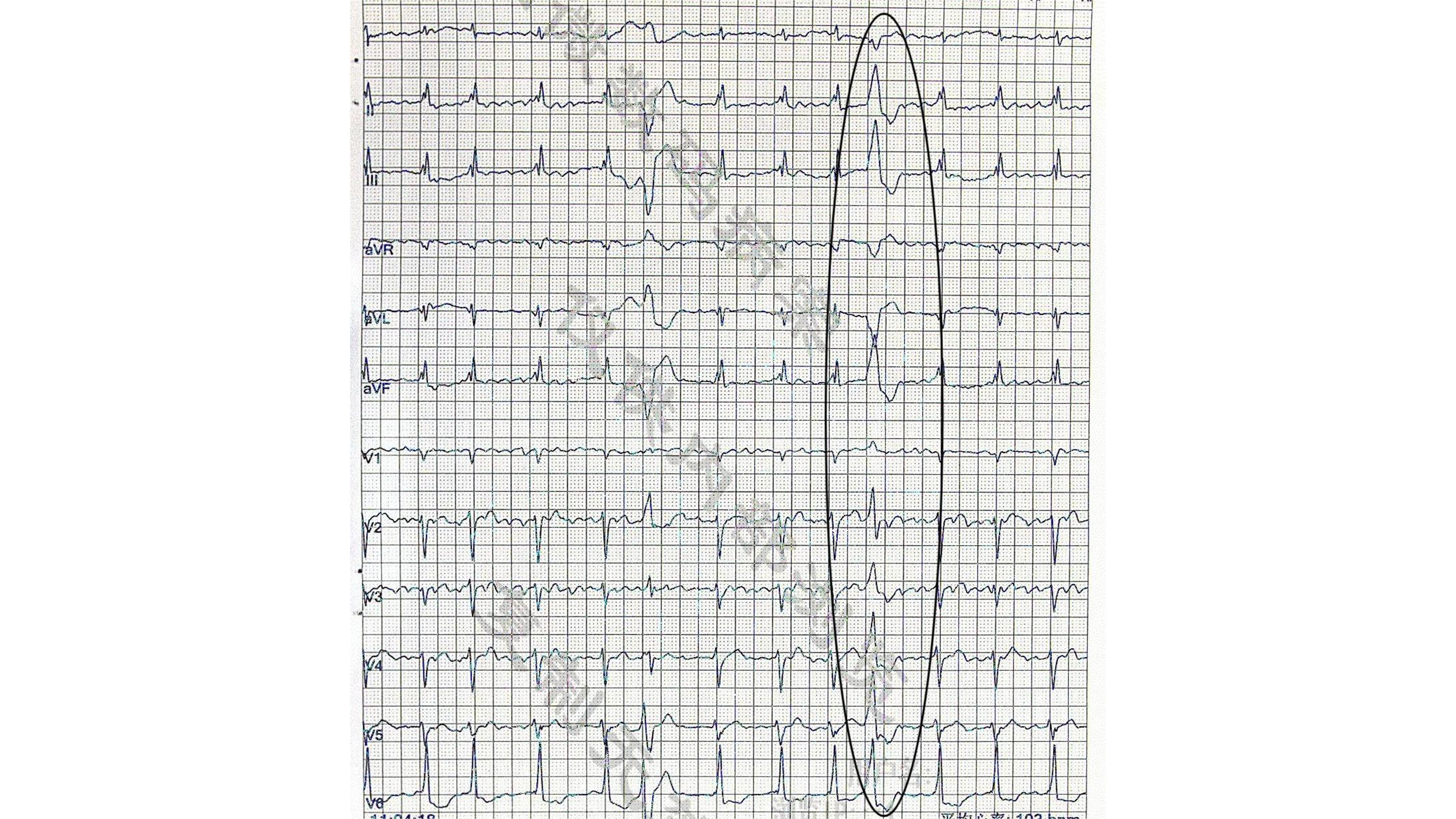

Supplement: ytaf166_Supplementary_Data [file ytaf166_supplementary_data.zip › supplemental 1a.PNG]

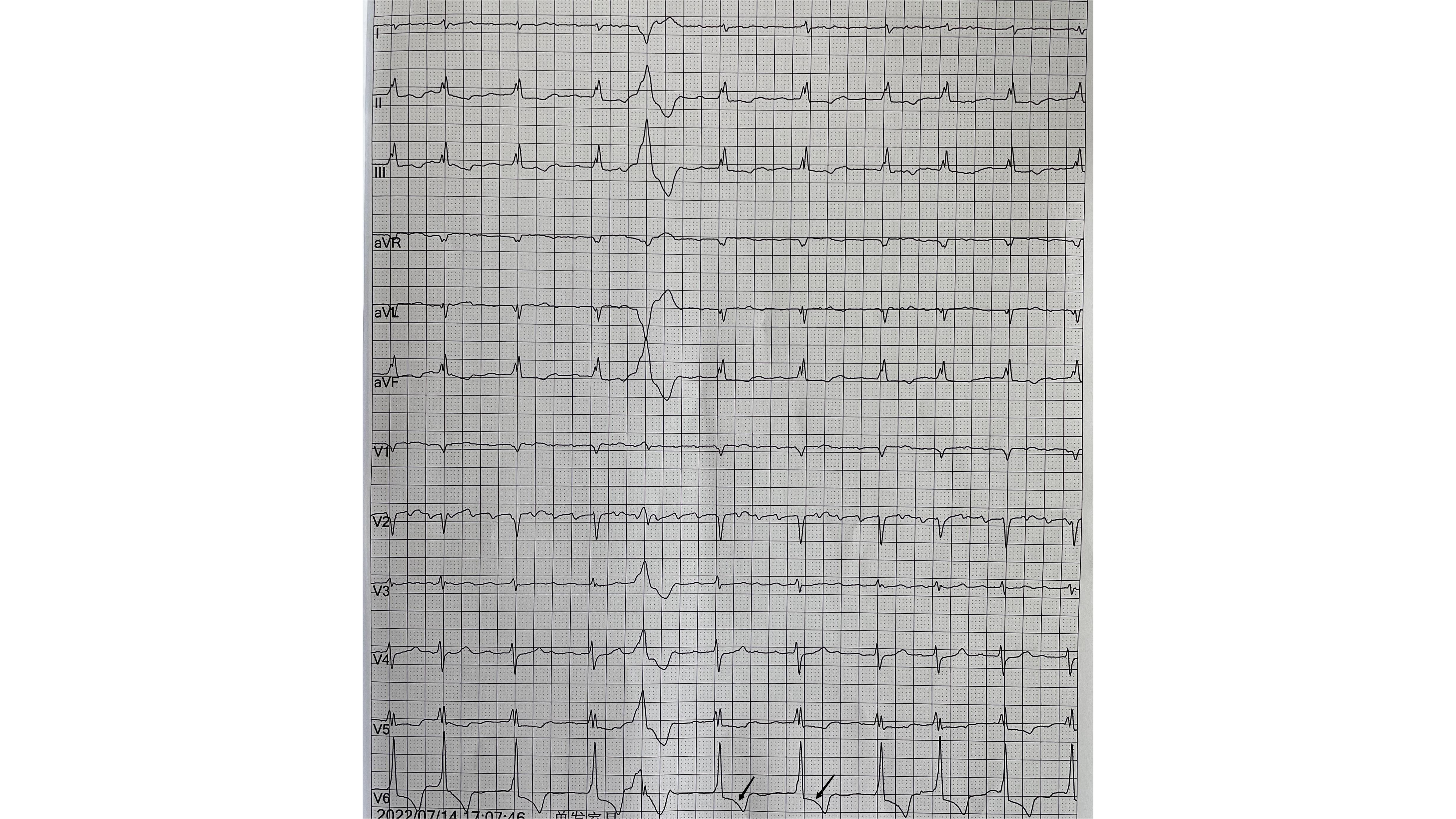

Supplement: ytaf166_Supplementary_Data [file ytaf166_supplementary_data.zip › supplemental 1b.PNG]

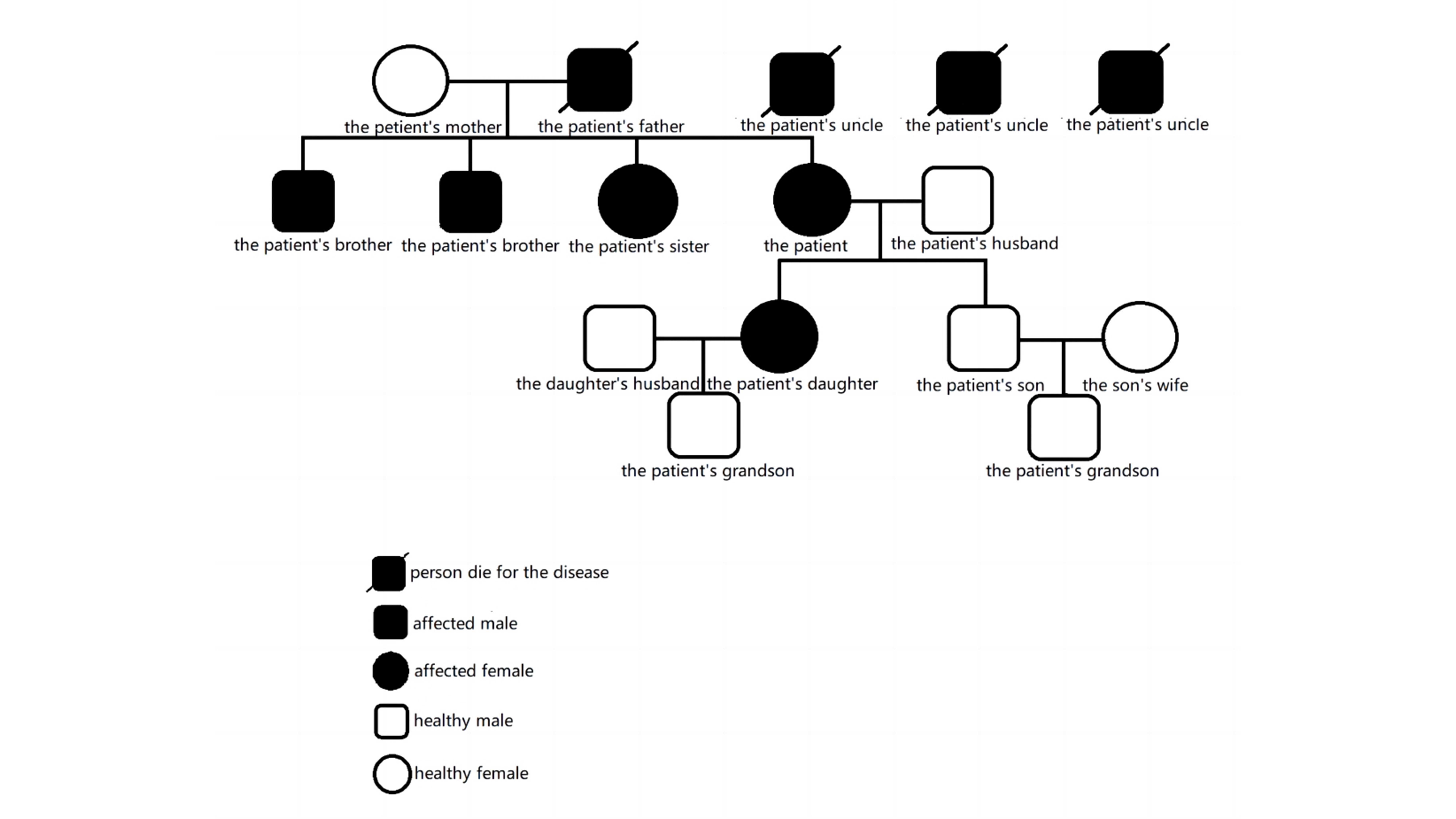

Supplement: ytaf166_Supplementary_Data [file ytaf166_supplementary_data.zip › supplemental 2.PNG]

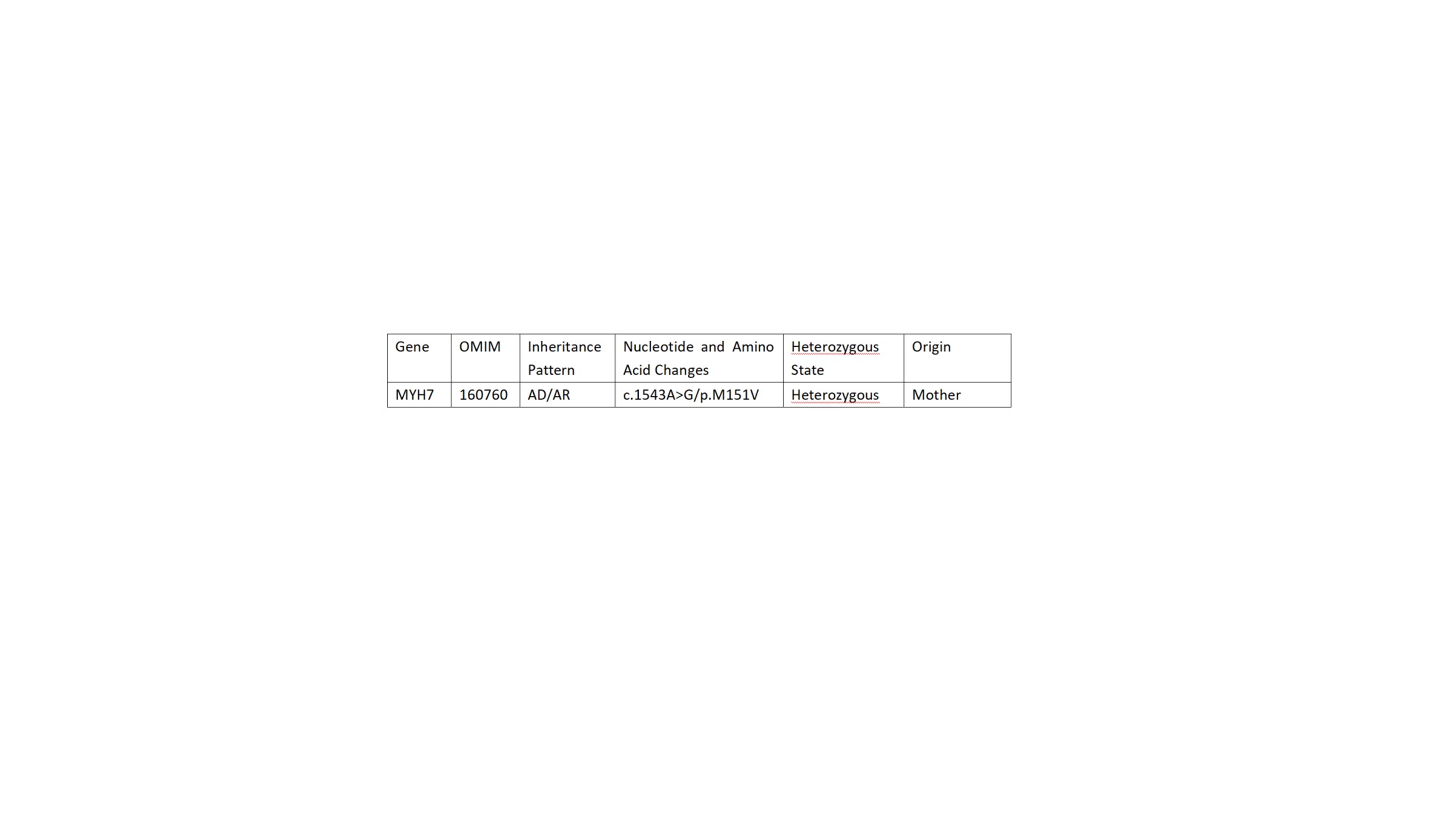

Supplement: ytaf166_Supplementary_Data [file ytaf166_supplementary_data.zip › supplemental 3.PNG]

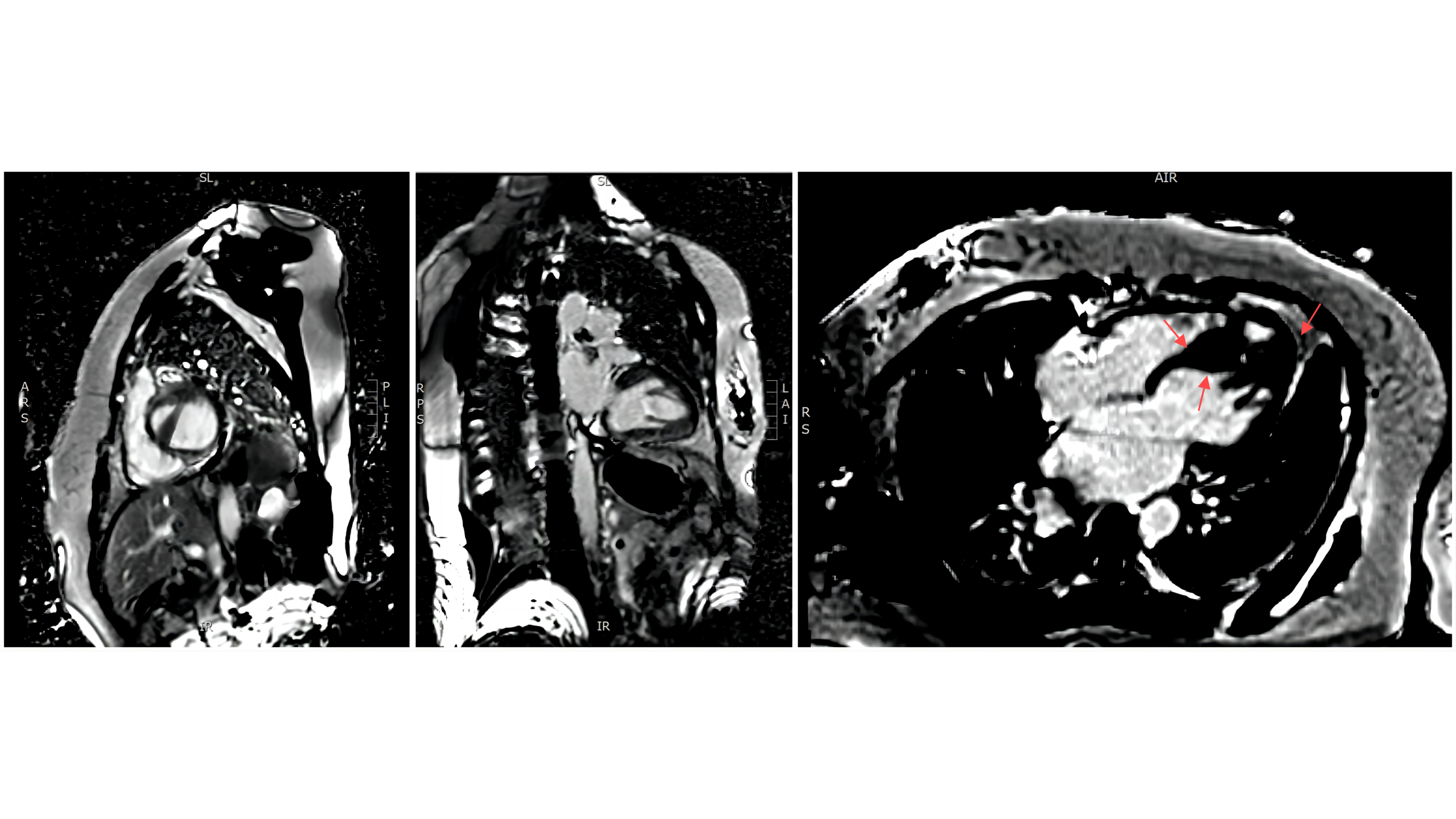

Supplement: ytaf166_Supplementary_Data [file ytaf166_supplementary_data.zip › supplemental 4.PNG]
